# Gait and frontal lobe function become associated in patients with idiopathic normal pressure hydrocephalus after shunt surgery

**DOI:** 10.3389/fneur.2025.1647707

**Published:** 2025-10-17

**Authors:** Masatsune Ishikawa, Etsuro Mori, Hiroaki Kazui

**Affiliations:** ^1^Department of Administraton, Rakuwa Villa Ilios, Kyoto, Japan; ^2^Normal Pressure Hydrocephalus Center, Otowa Hospital, Kyoto, Japan; ^3^Department of Behavioural Neurology and Neuropsychiatry, Osaka University United Graduate School of Child Development, Osaka, Japan; ^4^Department of Neuropsychiatry, Kochi Medical School, Kochi University, Nankoku, Japan

**Keywords:** hydrocephalus, aged people, gait, cognition, frontal lobe

## Abstract

**Objective:**

The association between gait and cognition in older adults is well-established. This study investigated the association between gait and cognition, especially in the frontal lobe, in patients with idiopathic normal pressure hydrocephalus (iNPH). Studies using cognitive assessment batteries often treat grading scores as continuous; however, these scores are inherently ordinal, which may lead to inaccurate results. To address this, a generalized additive model with an ordinal smoothing penalty was applied to analyze the data, including the ordinal scale.

**Methods:**

This supplementary study is based on an open-labelled clinical trial, SINPHONI-2, which was designed to evaluate the effectiveness of lumboperitoneal shunting in patients with probable iNPH. Gait and frontal lobe function were assessed using the Timed Up-and-Go (TUG) test and Frontal Assessment Battery (FAB), respectively. Sixty-nine patients with available pre- and postoperative data were selected.

**Results:**

Compared to the preoperative values, TUG time showed statistically significant improvement at 3,6, and 12 months after surgery. The FAB total score, analyzed as continuous data, became significant at 6 months postoperatively. When the FAB task scores were analyzed as ordinal data, TUG time was significantly associated with the “motor programming” and “conflicting instructions” tasks at 3, 6, and 12 months. The “environmental autonomy” task showed a significant association at 6 and 12 months, and the “verbal fluency” task at 12 months. In contrast, no association was found for the “conceptualization” and “inhibitory control” tasks. Analyses using a generalized additive model revealed that most relationships between TUG time and FAB task scores were linear, although some were non-linear.

**Conclusion:**

The associations between TUG time and FAB task performance gradually strengthened over the first year after surgery. FAB tasks involving hand movements were significantly associated with improvements in gait. This analytical approach can be applied to datasets with ordinal predictors in various research fields, such as grading scales used to assess disease severity.

## 1 Introduction

The association between gait and cognitive impairment in older adults is well-known (1). Although this association may reflect simple coexistence with aging, it may be related to a common underlying pathology (2). The frontal lobe plays a key role in gait control by processing cognitive input from other cortical areas, generating plans and programs for gait behavior, and exerting influence on the brainstem and spinal cord (3). Gait is no longer considered an automated motor activity that utilizes minimal high-level cognitive inputs. Increasing attention is being given to the complex neuropsychological influences on walking and the interactions between mobility control and related cognitive and behavioral functions (4).

Idiopathic normal pressure hydrocephalus (iNPH), a disease observed in older adults, affects gait, cognition, and continence (5, 6). Although it is well recognized as a treatable form of dementia (7), cognitive improvement following cerebrospinal fluid (CSF) shunt surgery is generally less pronounced than improvement in gait. Cognitive impairment in iNPH is typically characterized by reduced attention, slowed psychomotor speed, impaired verbal fluency, and deficits in executive function (8, 9). Frontal lobe symptoms can be assessed using the Frontal Assessment Battery (FAB), originally devised by Dubois et al. (10). Miyoshi et al. (11) reported that patients with iNPH had lower total FAB scores and verbal fluency task scores compared to those with Alzheimer’s disease. They concluded that frontal lobe function was impaired in iNPH and that cognitive deficits were closely associated with gait disturbances.

The FAB consists of six tasks. Each task is scored on a scale of 0 to 3, with 3 being the highest score. The total score (maximum score = 18) was originally defined as the sum of the individual task scores. However, each task score is ordinal rather than continuous. Therefore, mathematical operations such as addition, subtraction, multiplication, and division on ordinal scores do not yield strictly meaningful results, because the intervals between ordinal values are not necessarily equal or known. Liddell et al. (12) demonstrated that treating ordinal data as continuous can lead to low detection accuracy, distorted effect size estimates, and significantly inflated false positive rates. While analyses using ordinal data as outcomes are common, statistical methods that treat ordinal data as predictors remain relatively uncommon.

The recent development of the ordinal smoothing penalty has increased the reliability of ordinal independent variable (= predictor) assessments (13). A generalized additive model (GAM) is an extension of the commonly used generalized linear model (GLM). GAM handles nonlinear effects by replacing linear terms with a smooth function. GAM is well suited for analyzing longitudinal data with non-linear trends. Furthermore the combined use of GAM and ordinal smoothing penalty (14) allows statistical inference of ordinal predictors. Using this new statistical model, we studied the association between gait and Mini-Mental State Examination (MMSE) subitems in patients with iNPH and found that the number of significant subitems with gait increased within 1 year following surgery (15).

In the study, we examined whether the FAB subitems showed similar associations with gait as the MMSE subitems. We also evaluated the clinical significance of using a statistical approach designed for ordinal predictors.

## 2 Materials and methods

### 2.1 Patients

**Figure 1:**
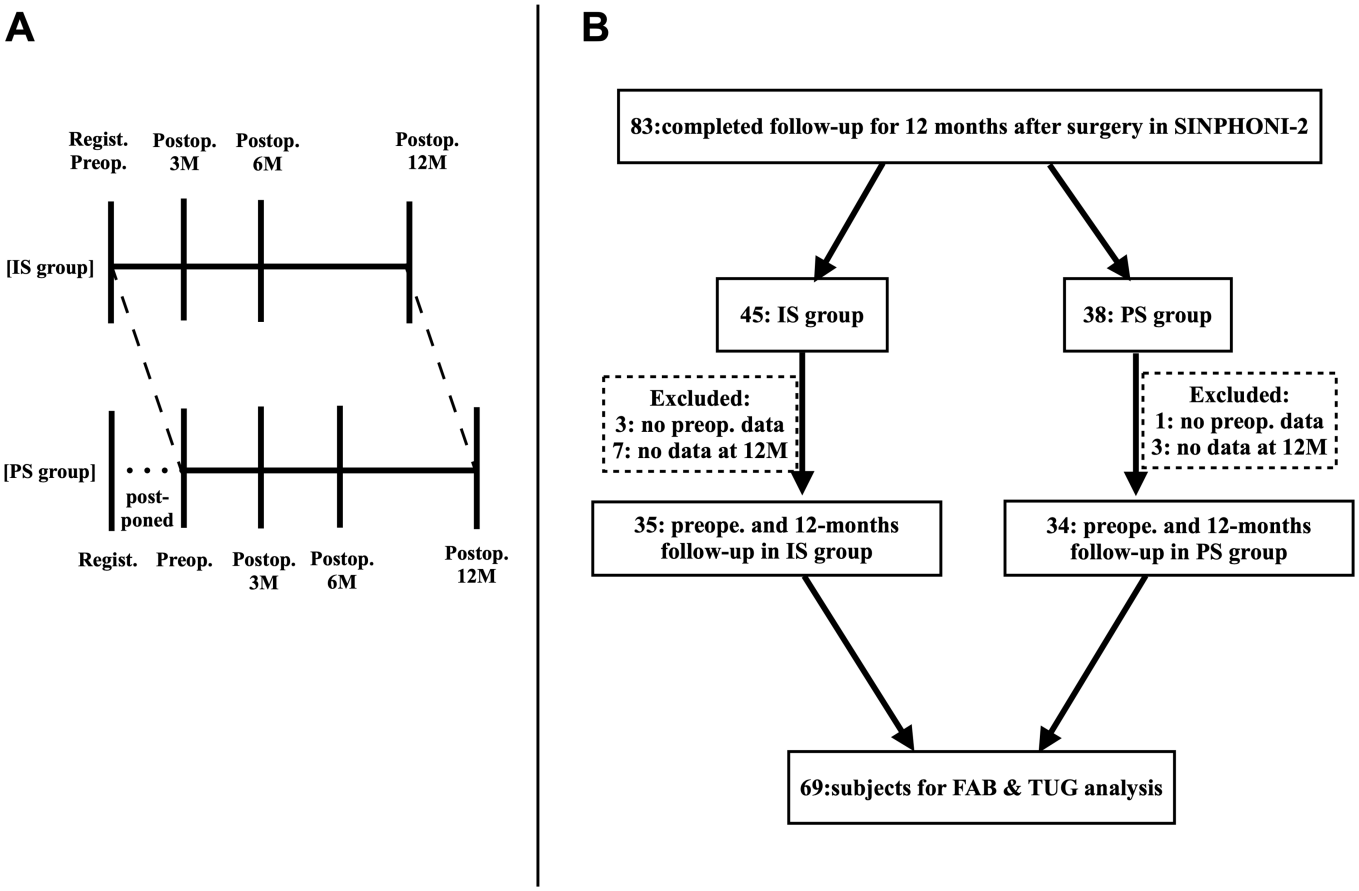
Study design of SINPHONI-2 **(A)**. In the PS group, surgery is postponed for 3-months. Both the IS and PS groups are followed up for 12 months after surgery. IS, immediate surgery; PS, postponed surgery, Regist., Registration; 3 M, 6 M, 12 M, 3, 6, and 12 months; SINPHONI-2, Study of Idiopathic Normal Pressure Hydrocephalus on Neurological Impairment-second study. Flowchart for patient selection **(B)**. Among the 93 patients included in this study, 83 complete the 12-month-follow-up after surgery (45 in the IS group and 38 in the PS group). Patients with data of the TUG and the FAB both at preoperative state and 12 months after surgery are selected, where a total of 69 patients are enrolled in this study: 35 patients in the IS group and 34 patients in the PS group.

The SINPHONI-2 trial is an open-label randomized trial (UMIN-CTR: UMIN000002730) that followed the Guidelines for Good Clinical Practice and adhered to the principles of the Declaration of Helsinki (2002) of the World Medical Association. The study protocol was approved by the Tohoku University Hospital Ethics Committee and the Institutional Ethics Committee of each participating institution. Written informed consent was obtained from all patients or their representatives. Details of the patients, definitions of iNPH, protocol compliance, and data collection (including data acquisition and management) have been described previously (16). The study protocol is described in the Supplementary material file. Briefly, 102 candidates diagnosed with possible iNPH according to the second edition of the Japanese iNPH guidelines (17) were recruited from 20 Japanese centers between March 2010 and October 2011. The inclusion criteria were as follows: age 60–85 years at the time of entry; presence of one or more NPH symptoms including gait disturbance, cognitive impairment, and urinary disturbance, assessed using the iNPH grading scale within 3 months before consent provision; ventriculomegaly with an Evans index >0.3; concurrent narrow sulci at high convexity; and an enlarged Sylvian fissure observed on computed tomography or magnetic resonance imaging. The exclusion criteria were as follows: the presence of secondary or congenital hydrocephalus or aqueductal stenosis, CSF pressure ≥20 cm H_2_O, complications of severe disuse muscle atrophy, and psychiatric disorders or other neurological diseases. According to these criteria, 93 patients were registered and randomly assigned to the immediate surgery (IS) or 3-month postponed surgery (PS) groups (Figure 1A). Randomization (1:1 ratio) was performed using a permuted block design with a block size of four or six within each clinical center according to a randomized code generated by the trial statistician. All patients in the IS group underwent lumboperitoneal shunt surgery using the Codman-Hakim programmable valve with the SiphonGuard (Codman Neuro-DePuy Synthes, Raynham, MA, USA). In the PS group, all patients underwent lumboperitoneal shunt surgery 3 months after registration. During the 3 months, patients in the PS group were instructed to perform physical tasks.

### 2.2 Assessment of gait and frontal lobe function

In SINPHONI-2 (Study of Idiopathic Normal Pressure Hydrocephalus on Neurological Improvement-second trial), gait performance was assessed in seconds using a 3-m timed Up-and-Go (TUG) test (18), which measures the time to complete the following six components: standing up from a chair, walking 3 m, turning around 180°, walking back to the chair, turning around 180° again, and sitting back down on the chair. All tests were performed twice. Because patients with severe conditions occasionally experienced interruptions in the second performance, data from the first performance were used. Frontal lobe function was assessed using the screening test developed by Dubois et al. (10). It consists of six tasks assessing the following executive functions: (1) conceptualization (similarities), (2) verbal fluency (mental flexibility), (3) motor programming (Luria’s fist-edge-palm (FEP) sequence (19)), (4) conflicting instructions (go-no-go test), (5) inhibitory control, and (6) environmental autonomy (prehension behavior). A modification was made to the “verbal fluency” task: the prompt “Say as many words as you can beginning with the letter ‘S’” was changed to “Say as many words as you can beginning with the letter ‘A’” because words starting with “S” are uncommon in Japanese (17).

### 2.3 Selection of patients in this study

Among the 102 potential patients, nine were eliminated owing to deviations from the inclusion criteria. Among the 93 patients included in this study, 83 completed the 12-month follow-up (45 in the IS group and 38 in the PS group). In the PS group, data obtained 3 months after registration were considered as the preoperative data. To examine the association between gait, assessed by TUG, and frontal lobe function, assessed by FAB, we selected patients with available preoperative and 12-month postoperative TUG and FAB data. In the IS group, three and seven patients were excluded due to the unavailability of preoperative and 12-month postoperative data, respectively. In the PS group, one and three patients were excluded due to the unavailability of preoperative and 12 months postoperative data, respectively. Consequently, 69 patients were enrolled in this study, 35 in the IS group and 34 in the PS group. Among them, one participant lacked 3-month postoperative data, and four lacked 6-month postoperative data (Figure 1B).

### 2.4 Statistical analysis

Among the SINPHONI-2 variables, age, sex, years of education, TUG test time (s), modified Rankin scale (mRS) scores (0–6), iNPH grading scale (0–4), and FAB total score were evaluated. The age and TUG test time were considered continuous variables. The FAB total score was considered a continuous variable based on the original work (10). The FAB task scores, as well as the mRS and iNPH grading scales, were treated as ordinal variables. These data were assessed at four different time points (preoperatively and at 3, 6, and 12 months postoperatively). In this dataset, there were one missing data point at 3 months and four at 6 months. We included these data for statistical analyses without imputation. All statistical analyses were performed using R software (20). Statistical significance was set at *p* < 0.05. For continuous variables, the mean and standard error were compared using parametric t-tests, following confirmation of normality with the Q-Q plots. Categorical data were analyzed using Fisher’s exact test because some categorical data had small sample sizes. Continuous data were plotted with means and standard errors using the “gggplot2” package (21). Categorical data, including ordinal data, were plotted as stacked bar plots. A multivariable linear model was applied with TUG test times as the dependent variable and FAB total scores as the independent variable, adjusting for age and years of education (both continuous covariates).

In contrast, FAB task scores were considered ordinal. Smoothing of the ordinal variables of the tasks was implemented using the “ordSmooth” function in the “ordPens” package (22), applying the smoothing penalty for adjacent dummy coefficients. The smoothed data were then analyzed with the GAM, using the “gam” function in the “mgcv” R package (14). Because the main interest was the relationship between TUG time and each FAB task, the GAM was applied to the respective tasks with age and years of education as covariates. GAM has the advantage of making inferences about associations between continuous outcomes and ordinal independent variables without placing parametric restrictions on the association (14). The *p*-values for ordinal data were reported only in cases of second-order penalties (13). The effective degrees of freedom (EDF) estimated from the GAM were used as proxies for the degree of nonlinearity in the relationships between the dependent and independent variables. An EDF of one is equivalent to a linear relationship. An EDF > 1 and ≤2 indicates a weak nonlinear relationship, while an EDF > 2 indicates a highly nonlinear relationship (23). Missing data, especially regarding years of education, were excluded from the multivariable linear regression and GAM.

## 3 Results

### 3.1 Demographic data

**Figure 2:**
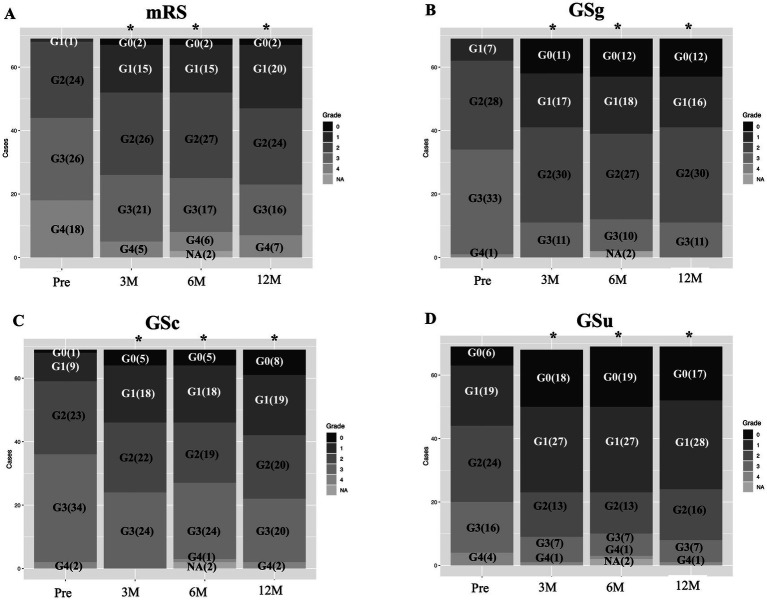
Sequential changes in general activity and NPH triad symptoms. In ADL assessed with mRS **(A)**, lower grades (less disability) increases after surgery. All triad NPH symptoms **(B–D)** assessed with the Japanese NPH grading scale-revised reveales statistically significant improvements. mRS, modified Rankin scale; GSg, GSc, GSu, iNPH grading scale of gait, cognition and urination; G0, G1, grade in respective grading scale; (), number of patients; Pre, preoperative state; 3 M, 6 M, 12 M, 3, 6, and 12 months after surgery; NA, not assessed.

Demographic data are listed in Table 1. In the IS group, data were obtained at registration and at 3, 6, and 12 months postoperatively. Data at the time of registration were considered preoperative data. In the PS group, preoperative data were collected 3 months after registration, just before the shunt surgery. The PS group showed lower mRS scores 3 months after registration compared to those at registration, but the difference was not statistically significant. Postoperative data were obtained in the IS group. Except for sex, preoperative differences in variables between the IS and PS groups were not statistically significant. Because our interest was focused on shunt effectiveness for possible iNPH, data from the IS and PS groups were combined for statistical analysis. Shunt responders (improvement by one or more points on the iNPH grading scale) were observed in 60.9, 53.6, and 55.1% of the cases, respectively, in gait, cognition, and urination. Sequential changes in the frequency of grades of general activity and NPH symptoms, assessed using the mRS and iNPH grading scales, respectively, are shown in Figure 2. Fisher’s exact test revealed that all parameters showed significant improvements in the postoperative state compared to the preoperative state. In this study, major co-morbidities were hypertension, hyperlipidemia, and diabetes. Their frequencies were 59.4, 33.3 and 26.01%, respectively. Correlation coefficients (r) with improvement on mRS at 1 year after surgery were 0.42, 0.08, and −0.03, respectively. For calculating the sample size of TUG time and FAB total, a power of 90% and a 2-sided alpha level of 0.05 were set for the analysis. Then, 55 patients were necessary for the TUG time, but 148 patients for the FAB total.

**Table 1:** Clinical characteristics of patients and comparison between the IS and PS groups.

Items	Total	IS group	PS group	p
Number of patients	69	35	34	IS vs. PS
Age (mean/SE)	77.0/0.6	77.1 /0.8	76.9/0.9	0.83
Sex, male (%)	55.1	40.0	70.6	0.02*
Education (mean/SE) (years)	12.5/0.5	12.1/0.7	12.9/0.7	0.36
TUG time (mean/SE) (seconds)	31.0/4.1	36.0/7.2	25.8/4.0	0.22
FAB total (mean/SE)	9.0/0.5	8.9 /0.7	9.2/0.7	0.79
Modified Rankin scale: grade 1 (cases)	0	0	0	0.33
Modified Rankin scale: grade 2 (cases)	19	12	7	
Modified Rankin scale: grade 3 (cases)	20	11	9	
Modified Rankin scale: grade 4 (cases)	11	7	4	

Except for sex, no parameters, including mRS score, shows statistically significant differences between the IS and PS groups. IS, immediate surgery; PS, postponed surgery; p, probability; FAB, frontal assessment battery; mRS, modified Rankin scale; SE, standard error; TUG, timed up and go test.

### 3.2 Sequential changes in TUG time and FAB total score, and their association

**Figure 3:**
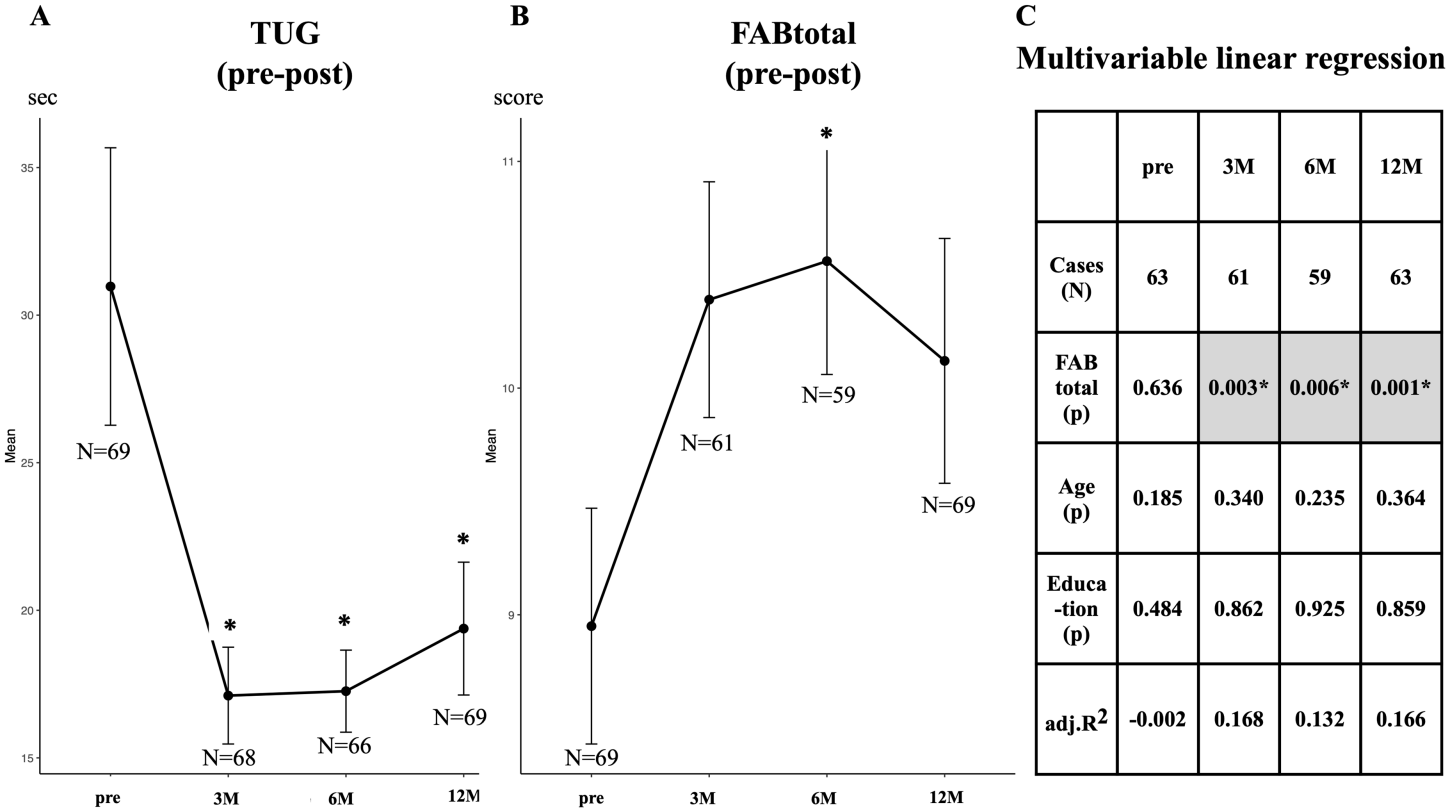
Sequential changes in TUG time and FAB total scores. TUG time **(A)** shows a statistical improvement at 3, 6, and 12 months after surgery. FAB total score **(B)** shows a significant improvement at 6 months. Regression analysis **(C)** with TUG as a responder and as a predictor of FAB total score reveales that both are statistically significant associations postoperatively. Age and years of education are covariables. Adj. R^2^, adjusted coefficient of determination; FABtotal, total score of Frontal Assessment Battery; p, probability; TUG, Timed Up and Go test.

A statistically significant decrease was observed in TUG at 3 months postoperatively, and its improvement continued at 6 and 12 months (Figure 3A). In contrast, the total FAB score increased after surgery; however, statistical significance was observed only at 6 months (Figure 3B). Multivariable linear regression analysis (Figure 3C) was performed with TUG test time as the dependent variable and FAB total score, with covariates of age and years of education as independent variables, all of which were continuous. No association was observed between TUG time and preoperative FAB total score. In contrast, statistically significant associations between them were observed at 3, 6, and 12 months after surgery.

### 3.3 Sequential changes in TUG time and FAB task score, and their association

**Figure 4:**
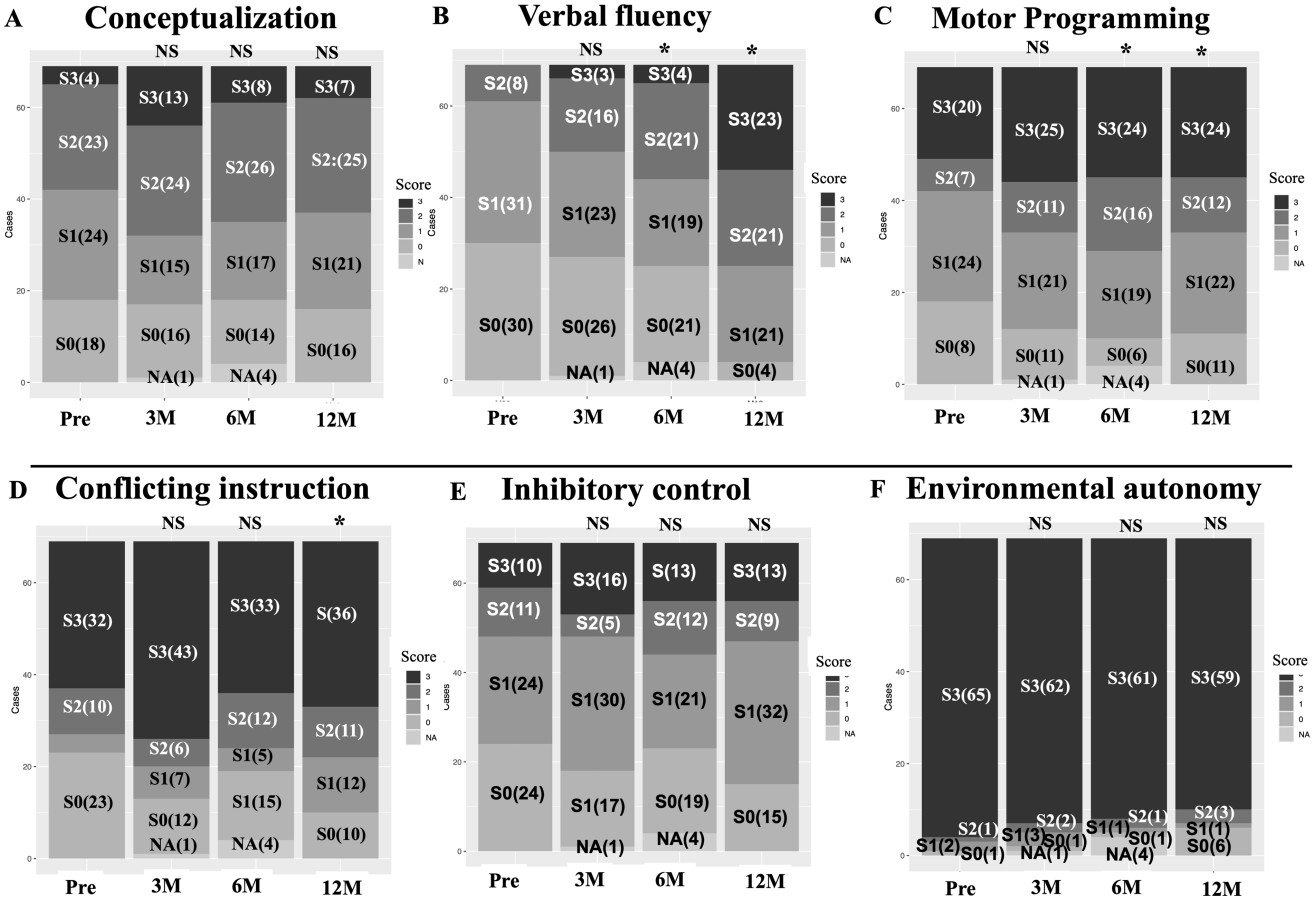
Sequential changes in the frequency of the FAB task scores. Pre-post comparisons of FAB tasks **(A–F)** using Fisher exact test shows statistically significant changes in “verbal fluency” **(B)**, “motor programming” **(C)** and “conflicting instructions” **(D)** in the postoperative follow-up periods. Note that most patients showed highest score (3 point) on the “Environmental autonomy” task **(F)**, both pre- and post-operatively. This suggests this task is easy to perform in them. Conversely, on the “conceptualization” and “inhibitory control” tasks **(A,E)**, a few patients show the highest score with relatively small variability, which suggests these tasks are difficult in most of patients. 3 M, 6 M, 12 M: 3, 6, and 12 months after surgery, NA, not assessed; p, probability; S, ordinal score of FAB subitems; (), number of patients.

Sequential changes in the frequency of the respective FAB task scores are shown in Figures 4A–F. Preoperative–postoperative comparison using Fisher’s exact test revealed that the “conceptualization,” “inhibitory control,” and “environmental autonomy” tasks (A, E, F) showed no statistical significance at 3, 6, and 12 months postoperatively. Conversely, the “verbal fluency” and “motor programming” tasks (B, C) showed statistically significant differences at 6 and 12 months postoperatively. The “conflicting instructions” task (D) showed statistical significance only at 12 months postoperatively. Only a few patients scored the highest score (3 points) on the “conceptualization” task (A) both preoperatively and postoperatively. This finding suggests that this procedure is difficult for elderly patients to perform. In addition, relatively few patients scored high scores (2 or 3 points) on the “inhibitory control” task (E). In contrast, most of the patients scored the highest for the “environmental autonomy” task (F) both preoperatively and postoperatively. This finding suggests that this procedure is easy for elderly patients to perform.

### 3.4 Association between TUG time and FAB task scores

**Figure 5:**
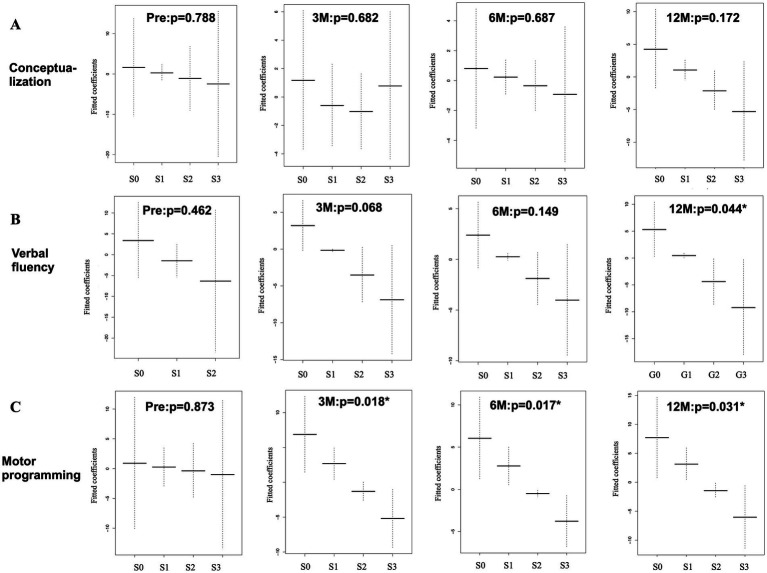
Association between TUG time and FAB ordinal task scores **(A–C)**, which was analyzed using GAM with an ordinal smoothing penalty. The “conceptualization” task **(A)** shows no statistically significant association with TUG time both at pre- and post-operative states. The “verbal fluency” task **(B)** shows a statistical significance at 12 months after surgery. The “motor programming” task **(C)** shows statistically significant associations at 3, 6, and 12 months. G: grading, p: probability, Pre: preoperative state, 3 M, 6 M, 12 M: 3, 6, and 12 months after surgery. Y-axis, fitted coefficients, dotted line: 95% confidence interval, X-axis, ordinal score(S); S0 to S3, *: statistical significance.

**Figure 6:**
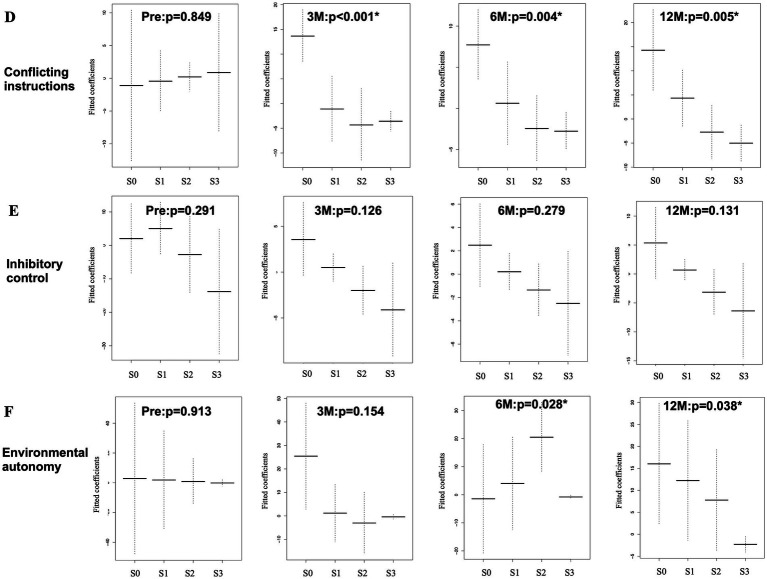
Association between TUG time and FAB ordinal task scores **(D–F)**, which was analyzed using GAM with an ordinal smoothing penalty. The “conflicting instructions” task **(D)** shows statistically significant associations at 3, 6, and 12 months. The “inhibitory control” task **(E)** shows no statistically significant association both at pre- and post-operative states. The “environmental autonomy” task **(F)** shows a statistically significant association at 12 months. Same abbreviations in Figure 5.

The association between TUG time and FAB task scores was examined for the six FAB tasks using the GAM. The sequential changes in the mean and standard errors are shown in Figures 5A–C, 6D–F. The y-axis shows the fitted coefficients of the respective task scores with the TUG time, and the x-axis shows the FAB scores. Because TUG time is inversely related to gait speed, lower coefficients for higher scores indicate a positive relationship between gait and frontal lobe function. The GAM results for this association are shown in Table 2. In the model, the responder was the TUG test time (continuous variable), and the predictors were the FAB task scores (ordinal variable), age (continuous variable), and years of education (continuous variable). Preoperatively, no statistically significant association was observed between the TUG test time and the six FAB task scores. At 3 months postoperatively, a statistically significant association was observed between both “motor programming” (C) and “conflicting instructions” (D) with gait. At 6 months postoperatively, a significant association was observed between “environmental autonomy” (F) and the TUG time, in addition to the previous two tasks. Twelve months postoperatively, “verbal fluency” (B) was statistically significant in addition to the previous three tasks. Therefore, a significant association was observed between both “motor programming” (C) and “conflicting instructions” (D) and TUG time at 3, 6, and 12 months postoperatively. The “environmental autonomy” (F) was statistically significant at 6 and 12 months postoperatively. The “verbal fluency” (B) was statistically significant only at 12 months postoperatively. Among the continuous predictors, only age at 3 months was significantly associated with the TUG time.

**Table 2:** Sequential changes of statistical significance for ordinal and continuous predictors on the association between TUG time and FAB tasks.

Time on assessment	Pre	3 M	6 M	12 M
Cases	63	61	59	63
(A) Conceptualization [EDF]	0.788 [1.001]	0.682 [1.5]	0.687 [1]	0.172 [1]
Age (estimate)	0.178 (1.290)	0.049* (0.681)	0.092 (0.484)	0.186 (0.622)
Education (estimate)	0.452 (−0.949)	0.569 (−0.259)	0.822 (−0.092)	0.278 (−0.674)
(B) Verbal fluency [EDF]	0.462 [1]	0.068 [1]	0.149 [1]	0.044* [1]
Age (estimate)	0.162 (1.306)	0.102 (0.553)	0.106 (0.455)	0.115 (0.714)
Education (estimate)	0.467 (−0.913)	0.640 (−0.208)	0.729 (−0.139)	0.309 (−0.621)
(C) Motor programming [EDF]	0.873 [1]	0.018* [0.052]	0.017* [1]	0.031* [1.001]
Age (estimate)	0.167 (1.317)	0.508 (0.241)	0.204 (0.351)	0.409 (0.393)
Education (estimate)	0.451 (−0.952)	0.798 (−0.112)	0.821 (−0.088)	0.431 (−0.482)
(D) Conflicting instructions [EDF]	0.849 [1]	<0.001* [2.18]	0.004* [1.781]	0.005* [1.66]
Age (estimate)	0.148 (1.380)	0.132 (0.438)	0.079 (0.463)	0.186 (0.580)
Education (estimate)	0.432 (−1.001)	0.624 (−0.185)	0.978 (−0.010)	0.625 (−0.286)
(E) Inhibitory control [EDF]	0.291 (1.697)	0.126 (1.147)	0.279 (1.211)	0.131 (1.17)
Age (estimate)	0.135 (1.375)	0.092 (0.573)	0.134 (0.430)	0.186 (0.612)
Education (estimate)	0.449 (−0.936)	0.739 (−0.150)	0.980 (−0.011)	0.484 (−0.440)
(F) Environmental autonomy [EDF]	0.913 [1.001]	0.154 [2.102]	0.028* [2.502]	0.038* [1.348]
Age (estimate)	0.151 (1.345)	0.055 (0.638)	0.094 (0.439)	0.100 (0.733)
Education (estimate)	0.470 (−0.935)	0.9997 (−0.002)	0.710 (−0.144)	0.966 (−0.028)

The number of statistically significant predictors in FAB (grayish cells) increases after surgery (3 M, 6 M, 12 M: 3, 6, and 12 months after surgery). The effective degrees of freedom (EDF) show that 1 is equivalent to linearity, and EDF > 2 is highly nonlinear. p, probability; SE, standard error; FAB, frontal assessment battery; TUG, timed up and go test. *, statistical significance.

### 3.5 Sequential changes of statistical significance for ordinal predictors on the association between TUG time and FAB task

Table 3 was extracted from Table 2 to summarize the sequential changes in the statistical significance of the ordinal predictors (A, B, C, D, E, and F). Statistically significant associations (gray cells) between the FAB tasks and TUG test time gradually increased after surgery. There was no significant association with the “conceptualization” (A) and “inhibitory control” (E) tasks. Although most of the EDFs for ordinal (non-linear) predictors were 1 or close to 1, three analyses (conflicting instructions at 3 months and environmental autonomy at 3 and 6 months) showed an EDF > 2, which indicated nonlinear relationships between them.

**Table 3:** Summary table of significant association between TUG time and FAB tasks across time points, analyzed using GAM with an ordinal smoothing penalty.

Time points	Pre	3 M	6 M	12 M
Cases	63	61	59	63
(A) Conceptualization [EDF]	0.788 [1.001]	0.682 [1.5]	0.687 [1]	0.172 [1]
(B) Verbal fluency [EDF]	0.462 [1]	0.068 [1]	0.149 [1]	0.044* [1]
(C) Motor programming [EDF]	0.873 [1]	0.018*[1.052]	0.017* [1]	0.031* [1]
(D) Conflicting instructions [EDF]	0.849 [1]	<0.001*[2.18]	0.004*[1.781]	0.005* [1.66]
(E) Inhibitory control [EDF]	0.291 [1.697]	0.126 [1.147]	0.279 [1.211]	0.131 [1.170]
(F) Environmental autonomy [EDF]	0.913 [1.001]	0.154 [2.102]	0.028*[2.502]	0.038*[1.348]

Statistical significance (*p* < 0.05) is indicated by * with a gray background in the cell. The tasks with statistical significance gradually increased after surgery. Three items showed EDF > 2, where two items showed statistically significant.

## 4 Discussion

In this study, we focused on the postoperative association between gait and frontal lobe function in patients with possible iNPH. Frontal lobe function was assessed using FAB. The association between TUG test time and FAB tasks was examined using the GAM. FAB task scores were regarded as ordinal independent variables. Preoperatively, no associations were observed between TUG time and FAB tasks. Postoperatively, the “motor programming” and “conflicting instructions” tasks showed statistically significant associations with gait at 3, 6, and 12 months. The “environmental autonomy” task showed statistical significance at 6 and 12 months postoperatively. The “verbal fluency” task showed significance at 12 months postoperatively. Therefore, the association between TUG time and FAB tasks gradually increased during the postoperative follow-up. No significant associations were observed for the “conceptualization” and “inhibitory control” tasks.

In this study, the TUG time was regarded as an assessment biomarker of gait. However, it also assesses dynamic balance, mobility, and fall risk. Thus, the term “gait” here was used in a broader sense.

A previous study using MMSE subitems found no statistically significant associations with TUG time preoperatively. However, subitems related to short-term memory, attention, and executive function became significant at 3 months after surgery. The number of statistically significant subitems gradually increased at 6 months and 12 months. The present study using FAB subitems showed that the “motor programming” and “conflicting instructions “were statistically significant at 3 months after surgery, with a similar trend of a gradual increase in the number of significant subitems over time. The “motor programming” task showed a statistically significant association with TUG time. It is plausible that improvements in motor programming function are closely related to improved gait after surgery in patients with iNPH. For the “motor programming” tas’s FEP sequence of hand motion (19) was applied in this study. Umetsu et al. (24) reported that functional magnetic resonance imaging revealed that the bilateral sensorimotor cortex, bilateral premotor and left parietal areas, ipsilateral cerebellum, contralateral sensorimotor area, and supplementary motor area (SMA) were activated during the FEP sequence. Using near-infrared spectroscopy (NIRS), Kobayashi et al. (25) revealed that the frontopolar and bilateral dorsolateral prefrontal cortices (DLPFC) are activated, especially during early trials of the FEP sequence. The SMA plays an important role in motor programming, while the DLPFC is closely related to working memory. Therefore, the improvement in the motor programming function associated with gait improvement suggests that shunt surgery activates wider areas of the frontal lobe and other cortical and subcortical structures.

The “conflicting instructions” task also showed a statistically significant association with TUG time. Of the FAB tasks, the “motor programming,” “conflicting instructions,” and “inhibitory control” tasks have the common characteristics of using working memory function and accompanying hand motion (26). In this study, the “motor programming” and “conflicting instructions” tasks showed a statistically significant association with TUG time after surgery, while the “inhibitory control” task did not show any association with TUG time. This difference may be explained by an investigation by Toyoda et al., who used NIRS (26). Toyoda et al. revealed that the “motor programming” and “conflicting instructions” tasks were simple enough for most adults to perform with few or no errors. Meanwhile, the score of the “inhibitory control” task in the healthy older group (60–81 years) was significantly lower than that in the younger group. They suggested that the older adults were more likely to make errors in the “inhibitory control” task than the younger adults, which was consistent with the decline in age with respect to the inhibitory control function reported by Hasher et al. (27). Our findings are consistent with these previous results, suggesting that task difficulty may also play a role in this association. In addition, the “conceptualization” task did not show any association with TUG time. This task had a poor correlation with gait performance. The “verbal fluency” task demonstrated a statistically significant association with TUG time at 12 months after surgery. This indicates that the association of the “verbal fluency” task with the TUG time is delayed compared to “motor programming” and “conflicting instructions” tasks. Therefore, the non-motor tasks, such as “conceptualization” and “verbal fluency” tasks, appeared to have a poor relationship with gait performance. These tasks may involve neural networks distinct from motor-related pathways, which may respond less effectively to shunt surgery.

The present and our previous studies indicate that both gait and cognition in patients with iNPH improve after surgery, suggesting the recovery of gait and cognition (some domains) using common neural networks. Furthermore, both studies showed that the number of significant associations increased during 12 months of follow-up. This suggests that improvements in gait may help reinforce beneficial cognitive circuits through shared neural networks. The delayed improvement in cognitive function, compared to gait, could be attributed to the slower recovery of functional networks involved in cognition. Recent discoveries of the glymphatic and meningeal lymphatic systems, responsible for clearing metabolic waste, may also be relevant to this delayed cognitive recovery (28, 29).

The frequency of the highest score (3 points) was high for the “environmental autonomy” task at all assessment time points, even in the preoperative state. This indicates that it is the easiest task among all the tasks. At 6 months, the EDF was 2.502, indicating a non-linear relationship. If a linear model were applied to examine the data, it would not be statistically significant.

A major strength of this study is that it demonstrates the usefulness of FAB task scores as ordinals on a scale using the GAM. Most FAB task scores in relation to TUG test time were considered linear; however, some were nonlinear (Table 3). Therefore, a recently developed statistical model with an ordinal smoothing penalty and GAM is useful and can be applied to continuous and non-continuous data on scales. This analysis can be applied to data involving ordinal predictors in various research fields, such as grading scales of disease severity. Second, the analysis of the respective FAB task scores provided more detailed information than the total FAB scores. This approach is also applicable to various fields of research, such as grading scales of disease severity. Third, most previous studies on the association between gait and cognition have reported impairments in both gait and cognition due to their coexistence or a common underlying pathology (1, 2). However, the present analysis revealed that shunt surgery improved gait and frontal lobe functions. Our previous study using the same SINPHONI-2 data revealed that the association between immediate memory assessed by MMSE subitem and gait was statistically significant at 3 months after surgery and continued for up to 12 months, whereas a statistically significant association between recall and gait was not observed during 12 months after surgery (15).

This study has some limitations. First, the sample size was small. To overcome this limitation, we investigated the relationship between gait and cognition in patients with possible iNPH, combining the IS and PS groups. Thus, 69 patients who completed the 12-month follow-up, including assessments of TUG time and FAB tasks, were selected for analysis. These patients were divided into immediate and delayed surgical groups, although the overall sample size remained small. Additionally, potential learning effects on the FAB and the absence of a non-surgical control group are important limitations to consider. In SINPHONI-2, there was no statistically significant difference between the two groups at 1 year postoperatively, indicating that a 3-month delay in surgery can reverse symptoms to almost the same degree as in the immediate surgery group. Third, some patients included in this study may have had coexisting vascular dementia (30) or Alzheimer’s disease (31), which could have negatively impacted the results. However, shunt surgery may improve frontal subcortical ischemia, potentially leading to favorable outcomes despite these comorbidities. Fourth, no control group was included. As this study is a supplementary study of SINPHONI-2, all participants who met the eligibility criteria underwent shunt surgery. To exclude the learning effect of the tasks, sequential changes in TUG time and FAB task score would be necessary in a group of patients with probable iNPH who did not undergo surgery. However, it is ethically difficult not to operate on patients with the possibility of symptomatic improvement after shunt surgery.

## 5 Conclusion

This study demonstrated a gradual increase in the statistically significant association between gait and cognition, particularly frontal lobe function, after shunt surgery in patients with iNPH, as assessed using the FAB tasks. Tasks involving hand movements showed a strong association with gait improvement. Furthermore, analyzing the FAB task scores as ordinal data provided more detailed information than analyzing the total FAB score as continuous data.

## Data Availability

The datasets presented in this study can be found in online repositories. The names of the repository/repositories and accession number(s) can be found in the article/Supplementary material.
